# Effectiveness of sodium bicarbonate infusion on mortality for elderly septic patients with acute metabolic acidosis

**DOI:** 10.3389/fphar.2022.974271

**Published:** 2022-09-13

**Authors:** Sai Huang, Yaojun Peng, Lili Wang, Jing Wang, Xuan Zhou, Xiang Cui, Li Chen, Bo Yang, Cong Feng

**Affiliations:** ^1^ Department of Hematology, Fifth Medical Center of Chinese PLA General Hospital, Beijing, China; ^2^ National Clinical Research Center of Geriatric Diseases, Chinese PLA General Hospital, Beijing, China; ^3^ Department of Emergency, First Medical Center of Chinese PLA General Hospital, Beijing, China; ^4^ Department of General Medicine, First Medical Center of General Hospital of People’s Liberation Army, Beijing, China; ^5^ Department of Emergency, Hainan Hospital of Chinese PLA General Hospital, Sanya, China; ^6^ Department of Orthopedics, Fourth Medical Center, General Hospital of People’s Liberation Army, Beijing, China; ^7^ Department of Thoracic Surgery, First Medical Center, General Hospital of PLA, Beijing, China; ^8^ State Key Laboratory of Kidney Diseases, National Clinical Research Center of Kidney Diseases, General Hospital of People’s Liberation Army, Beijing, China; ^9^ Department of General Medicine, Hainan Hospital of Chinese PLA General Hospital, Sanya, China

**Keywords:** early sodium bicarbonate infusion, old patient, sepsis, mortality, acute metabolic acidosis

## Abstract

**Objective:** This study aimed to explore the effectiveness of sodium bicarbonate (SB) infusion on mortality in elderly septic patients with acute metabolic acidosis (MA) and in other subgroups.

**Methods:** Retrospective analysis of a large ICU database (MIMIC-IV) was performed. Elderly septic patients with acute MA were identified from MIMIC-IV. Propensity score analysis (PSA) was performed to explain for the baseline differences in the probability to receive SB or not. The marginal structural Cox model (MSCM) was developed to adjust for both baseline and time-varying confounding variables. The primary outcome was the ICU and hospital mortality.

**Results:** A total of 869 elderly septic patients with acute MA were identified in this study, including 361 in the SB group and 508 in the non-SB group. In the PSA, SB infusion was not associated with reduced ICU (HR 0.82, 95% CI 0.62–1.10; *p* = 0.19) or hospital (HR 0.94, 95% CI 0.74–1.19; *p* = 0.60) mortality in overall elderly septic patients with acute MA. In the subgroup of severe metabolic acidosis, SB infusion could not improve the ICU (HR 0.82, 95% CI 0.62–1.10; p =0.19) and hospital (HR 0.94, 95% CI 0.74–1.19; p =0.60) mortality on elderly septic patients. However, In the subgroup of moderate metabolic acidosis, SB infusion could be found associated with improved ICU (HR 0.64, 95% CI 0.43–0.95; *p* <0.05) and hospital (HR 0.70, 95% CI 0.50–0.99; *p* <0.05) survival in elderly septic patients. In the MSCM, the results were similar with PSA.

**Conclusion:** SB infusion could improve both ICU and hospital survival for elderly septic patients with acute metabolic acidosis.

## Introduction

Sepsis is widely recognized in intensive care units. With more patients being diagnosed with severe sepsis, the total number of sepsis-related deaths is increasing (Vincent and Abraham, 2006). Sepsis is also a common cause of hospitalization in the elderly as more than half of the cases presented are over the age of 65 years (Skogberg et al., 2012; Yahav et al., 2016). A longitudinal observational study revealed that although older people represent only about 12% of the American population, 65% of the sepsis cases are older patients. Compared to younger patients, this yields a relative risk of death (Martin et al., 2006). The clinical outcomes of sepsis in older adults are worse than in their younger counterparts due to higher rates of organ failure and mortality (Lee et al., 2007; Wester et al., 2013; Englert and Ross, 2015). Elderly septic patients were reported shorter durations of hospitalization before death and those who survived severe sepsis were more likely to be transferred to specialized care facilities than returning home (Martin et al., 2006). A recent prospective multicenter study involved 1,490 elderly patients showed that prompt treatment within the first 6 h could reduce mortality rate of sepsis in elderly patients (Wernly et al., 2020), suggesting that management is critical to survival for elderly septic patients.

Metabolic acidosis (MA) is frequently found in patients with sepsis. It is one of the most important factors associated with mortality. Bernhard Wernly et al. have reported that acidosis is an independent predictor from hyperlactatemia for septic mortality (Wernly et al., 2020). Sodium bicarbonate (SB) infusion is mostly administered for reversal of severe metabolic acidosis (SMA), which could improve the acidotic status (Kraut and Kurtz, 2006; Parker and Parshuram, 2013). A retrospective observational study has revealed that SB infusion may reduce ICU mortality in septic patients presented with AKI stage 2 or 3 and SMA (11). Therefore, SB administration as one of the early managements for MA may improve the survival of the elderly septic patients.

Base on the above context, this investigation aims to further explore the clinical effectiveness of SB infusion on septic mortality in completely elderly patients with acute MA and in other subgroups. Considering that SB infusion is a time-weighted predictor which relies on the bicarbonate concentration (BC) and blood pH, marginal structural Cox model (MSCM) has been developed to explain baseline data, time-weighted covariates and records of SB infusion (Robins et al., 2000; de Keyser et al., 2014; Karim et al., 2014).

## Methods

### Study design and database

The current investigation is a retrospective analysis using a large database (MIMIC-IV,v0.4) that included 76540 critically ill admissions between 2008 and 2019 among 6 ICUs from a single center hospital (Beth Israel Deaconess Medical Center, BIDMC; Boston, Massachusetts) (Johnson et al., 2016; Johnson et al., 2018; Johnson et al., 2020). Because the database we analyzed here had already gained approval from an institutional review board (IRB) and been available to the public, exemption from additional institutional IRB approval was allowed.

### Study cohort

The current study included all admissions from the MIMIC-IV. However, only the recorded first ICU admissions were analyzed (Zhang et al., 2018). Inclusion criteria: 1) age ≥ 60 years old; 2) had sepsis on ICU admission; 3) had acute MA (MA occurs within a few days (Yagi and Fujii, 2021)) with pH less than 7.3 and BC less than 20 mmol/l; 4) without respiratory acidosis (PaCO2 less than 50 mmHg) (Zhang et al., 2018). Patients’ pH, BC and PaCO2 were recorded within 48 h after ICU admission. The third International Consensus Definitions defined sepsis as a condition with “life-threatening organ dysfunction caused by a dysregulated host response to infection” (Singer et al., 2016). If multiple test results were recorded, only the maximum value of PaCO2, and the minimum values of BC and pH were enrolled. Patients who were treated in the ICU for more than 100 days, and those with cardiac arrest were excluded (Zhang et al., 2018).

### Variables

The selected variables from the MIMIC-IV database were data recorded within 24 h after that patients were admited to ICU, which comprised gender, age, body mass index (BMI), comorbidities on ICU admission, mechanical ventilation, vasopressors and renal replacement therapy (RRT). Following the guidelines of the Kidney Disease: Improving Global Outcomes (KDIGO), patients with acute kidney injury (AKI) were identified (Kellum and Lameire, 2013). The state of shock during the first day after ICU entry was identifined by shock index (shock index >1.0) (Allgöwer and Burri, 1967). AKI stages were identified by serum creatinine within the first 48 h of ICU stay. The infusion of lactate solution for the first 24 h was enrolled for investigation. The year of admission was included as a random factor in mixed-effect modeling (SerpaNeto et al., 2018).

Laboratory variables of PaCO2, pH, BC and lactate were recorded through the whole ICU period. Data extraction from the MIMIC-IV included both the laboratory analysis result of physiological values and the time on the lab chart. For patients who were tested multiple times, the highest value of lactate and PaCO2, and the lowest value of BC and pH each day were enrolled for investigation.

### Outcomes

The primary outcome was patient mortality in the hospital and ICU. Both were identified as the outcomes of patients survival on discharge from hospital and ICU.

### Statistical analysis

According to patients’ SB infusion status within the first 48 h of ICU stay, the investigation cohort was categorized into two groups, intervention (SB) group and control (non-SB) group. The continuous data was reported as mean with standard deviation or median with interquartile range (IQR) accordingly. Student’s t-test or Mann-Whitney U test was implemented accordingly to determine if there were any significant differences between two groups. Categorical variables were reported as absolute frequency (n) and relative frequency (%). Accordingly, either chi-square test or Fisher’s exact test was performed for data analysis.

Propensity score analysis (PSA) was used to explain baseline differences of the probability of an admission that would receive SB infusion treatment (Zhang, 2017). In the PSA, the SB group were treated with SB infusion within the first 48 h after ICU admission. The variables used in PSA were list in Supplementary Tables S4, S5. Nearest neighbor method was used for propensity score matching between SB group and non-SB group. Last, residual imbalance was adjusted in Cox regression model by including parameters with p values less than 0.05, and clinical expertise used in judgement of any potential confounders.

SB infusion treatment during hospitalization in the ICU was treated as a time-weighted variable when it was analyzed in MSCM. Variables such as gender, age, BMI, comorbidities, use of mechanical ventilation as well as vasopressor and RRT were extracted as potential baseline confounders. These items were recorded within the first 24 h after patients were admitted to the ICU. Laboratory variables such as BC, PaCO2, lactate and pH throughout the period of ICU stay were enrolled as time-varying confounders in the model. Inverse probability weighting estimation in the R (version 4.0.0) and “ipw” package (version 1.0-11) were performed to MSCM parameters for correcting confounding and selection bias formed by informative censoring (Robins et al., 2000).

Missing values (<25%) were identified in four items, pH, PaCO2, BMI, BC and lactate (Supplementary Table S1). Multiple imputation procedure was applied to these variables with missing values. Multiple imputation was conducted using the method of predictive mean matching for continuous variable, logistic regression for categorical variables, and five databases were created. The multivariable model was reproduced in the five databases after multiple imputation and the results were pooled (SerpaNeto et al., 2018). To avoid bias introduced by missing data, and assuming that data were missing at random, the analysis of the primary outcome was replicated after multiple imputation before PSA procedure.

The subgroup investigations were further processed in both PSA and MSCM analyses by categorizing patients into (Vincent and Abraham, 2006) SMA (pH < 7.2, BC <20 mmol/l and PaCO2<50 mmHg) and (Yahav et al., 2016) moderate metabolic acidosis (MMA, 7.2 ≤ pH < 7.3, BC <20 mmol/l and PaCO2<50 mmHg) (Zhang et al., 2018; Yagi and Fujii, 2021).

R package (version 4.0.0) was used in this study to perform all statistical analyses. To be considered having statistically significant difference, the cut-off point for p value was less than 0.05.

## Results

### Study cohort

**FIGURE 1:**
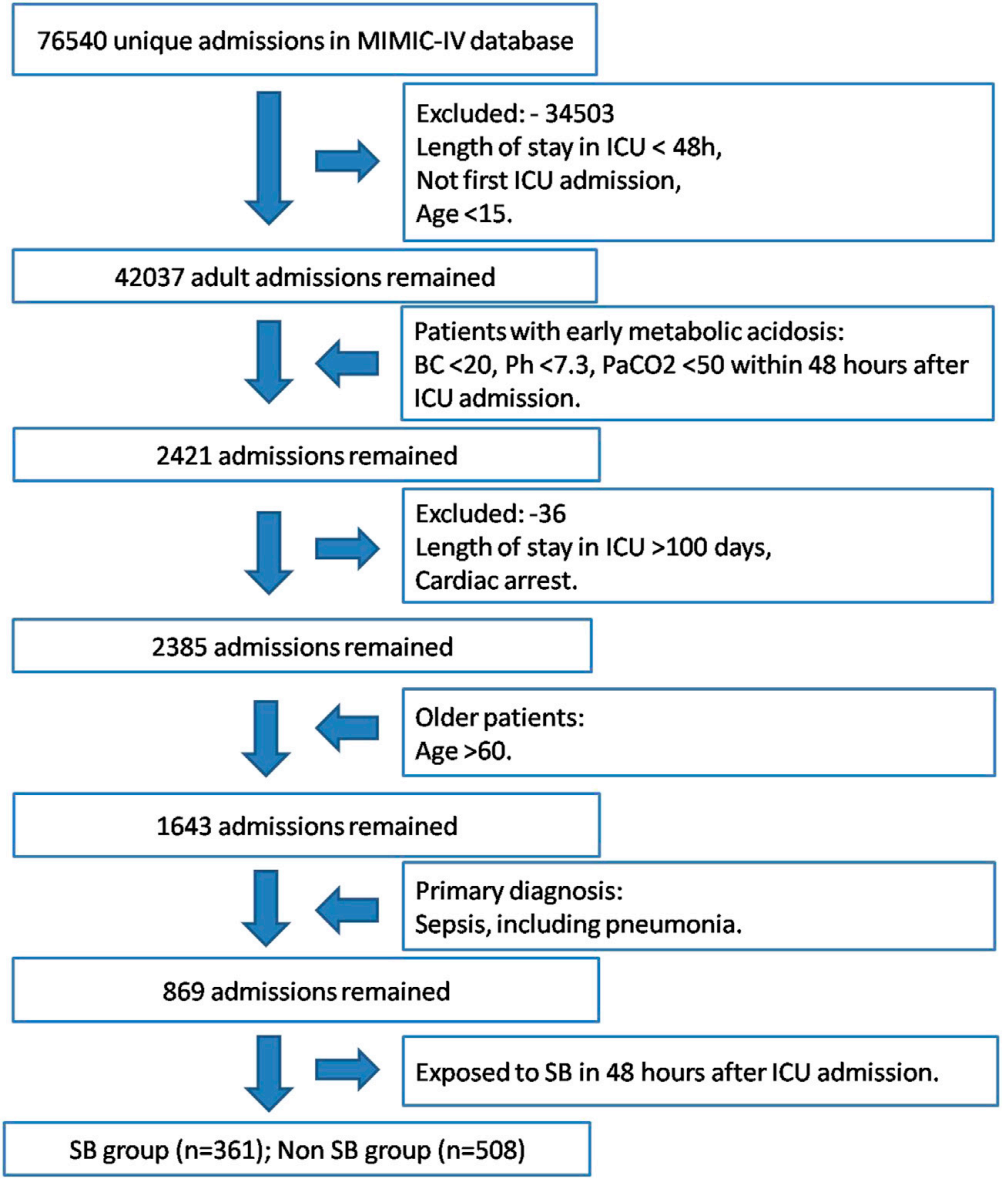
Study cohort selection workflow of MIMIC-IV database based on the designed inclusion and exclusion criteria.

A number of 869 patients were identified as acute MA within the first 48 h after ICU admission from the MIMIC-IV. Among the study population, 361 patients received SB infusion treatment in the first 48 h of ICU stay, and 508 patients didn’t receive this treatment (Figure 1).

Table 1 revealed the baseline information for SB group and non-SB group. Patients in the SB group were reported with lower percentage of hypertension [145 (40.17%) vs. 241 (47.44%); *p* < 0.05] than those in the non-SB group. During the first 24 h after being admitted to ICU, SB group revealed higher incidence of severe AKI (AKI stages 2 or 3) [73 (20.22%) vs. 32 (6.30%); *p* < 0.01] and shock [273 (75.62%) vs. 326 (64.17%); *p* < 0.01], and were more likely to have vasopressors [315 (87.26%) vs. 412 (81.10%); *p* < 0.05] and RRT [87 (24.10%) vs. 34 (6.69%); *p* < 0.01] than the non-SB group. No statistically significant difference was found in using mechanical ventilation between the two groups. The results of pH [7.16 (0.14) vs. 7.23 (0.08), *p* < 0.01] and BC [13.00 (5.00) vs. 17.00 (4.00), *p* < 0.01] were significantly lower, and of lactate were significantly higher [5.80 (7.00) vs. 3.00 (3.00), *p* < 0.01] in the SB group than those in the non-SB group. Lactate solution usage was statistically higher in the non-SB group [236 (46.46%) vs. 142 (39.34%); *p* < 0.01] than that in the SB group. No statistically significant difference was found during admission period between the two groups. No patient with multiple admissions was recorded in the cohort.

**TABLE 1:** Baseline differences between two groups before propensity score matching.

Key characteristics	Non-SB group (n)	SB group (n)	P-value
Demographic information:			
Final cohort (n)	508	361	—
Age, years (median, (IQR))	75.00 (17.00)	73.00 (15.0)	0.15
Male gender (n (%))	261 (51.38)	185 (51.25)	1.00
BMI, (median, (IQR))	28.37 (8.74)	28.17 (8.61)	0.87
Admission period, n(%)			0.64
Before 2014	323 (63.58)	236 (65.37)	—
2014-2019	185 (36.42)	125 (34.63)	—
Comorbidities (n(%)):			
Hypertension	241 (47.44)	145 (40.17)	<0.05
Diabetes	187 (36.81)	140 (38.78)	0.60
Congestive heart failure	183 (36.02)	128 (35.46)	0.92
Chronic pulmonary disease	81 (15.94)	50 (13.85)	0.45
Chronic kidney disease	159 (31.30)	129 (35.73)	0.20
Chronic liver disease	18 (3.54)	19 (5.26)	0.29
The incidence of AKI stage 2 or 3 and shock status, (n (%)):			
AKI-23	32 (6.30)	73 (20.22)	<0.01
AKI-3	31 (6.10)	71 (19.67)	<0.01
AKI-2	1 (0.20)	2 (0.55)	0.77
Renal replacement therapy	34 (6.69)	87 (24.10)	<0.01
Shock	326 (64.17)	273 (75.62)	<0.01
Additional respiratory and hemodynamic support, (n (%)):			
Mechanical ventilation	427 (84.06)	302 (83.66)	0.95
Vasopressors	412 (81.10)	315 (87.26)	<0.05
Laboratory values, (median, (IQR)):			
Minimum PaO2, %	82.00 (34.00)	74.00 (30.00)	<0.01
Maximum PaCO2, %	44.00 (9.25)	43.00 (14.00)	0.28
Minimum pH	7.23 (0.08)	7.16 (0.14)	<0.01
Minimum bicarbonate concentration, mmol/L	17.00 (4.00)	13.00 (5.00)	<0.01
Maximum lactate, mmol/L	3.00 (3.00)	5.80 (7.00)	<0.01
Lactate solution, (n (%))	236 (46.46)	142 (39.34)	<0.05

AKI, acute kidney injury; AKI-2, AKI stage 2; AKI-3, AKI stage 3; AKI-23, AKI stage 2 or 3. AKI stages were based on serum creatinine criteria of KDIGO (Kidney Disease: Improving Global Outcomes).

### Outcomes of propensity score analysis

The SB group with 361 patients were matched with the same number of patients from the non-SB group in PSA. The imbalance between the two groups was significantly improved after PSA (Supplementary Table S2). Cox proportional hazard model was developed because there were still residual imbalances between the two groups. The results revealed that there was no association between SB infusion and reduced ICU (HR 0.82, 95% CI 0.62–1.10; *p* = 0.19) or hospital (HR 0.94, 95% CI 0.74–1.19; *p* = 0.60) mortality in overall elderly septic patients with acute MA (Table 2, Table 3). In the subgroups, the ICU and hospital mortality effects on elderly septic patients with acute SMA had not found any statistically significant difference. However, SB infusion was found to be associated with improved ICU (HR 0.64, 95% CI 0.43–0.95; *p* < 0.05) and hospital (HR 0.70, 95% CI 0.50–0.99; *p* < 0.05) survival in elderly septic patients with acute MMA. (Tables 2–5).

**TABLE 2:** Association of sodium bicarbonate infusion and ICU mortality in the overall and subgroups by using propensity score analysis.

Overall and subgroups	Hazard ratio	Lower.95	Upper.95	P Value
Overall population (n = 869)	0.82	0.62	1.10	0.19
Severe metabolic acidosis (n = 274)	1.97	0.90	4.31	0.09
Moderate metabolic acidosis (n = 595)	0.64	0.43	0.95	<0.05

**TABLE 3:** Association of SB infusion and hospital mortality in the overall and subgroups by using propensity score analysis.

Overall and subgroups	Hazard ratio	Lower.95	Upper.95	*p* Value
Overall population (n = 869)	0.94	0.74	1.19	0.60
Severe metabolic acidosis (n = 274)	1.98	0.97	4.03	0.06
Moderate metabolic acidosis (n = 595)	0.70	0.50	0.99	<0.05

**TABLE 4:** Cox regression model after propensity score matching for predicting ICU mortality in patients with sepsis and acute moderate metabolic acidosis.

Variables	Hazard ratio	Lower.95	Upper.95	P Value
Use of sodium bicarbonate	0.64	0.43	0.95	<0.05
Mechanical ventilation	0.59	0.34	1.00	<0.05
Minimum bicarbonate concentration	0.90	0.82	0.99	<0.05

**TABLE 5:** Cox regression model after propensity score matching for predicting hospital mortality in patients with sepsis and acute moderate metabolic acidosis.

Variables	Hazard ratio	Lower.95	Upper.95	P Value
Use of sodium bicarbonate	0.70	0.50	0.99	<0.05
Chronic pulmonary disease	1.68	1.08	2.63	<0.05
Minimum bicarbonate concentration	0.92	0.85	0.99	<0.01

### Outcomes of marginal structural cox model

**FIGURE 2:**
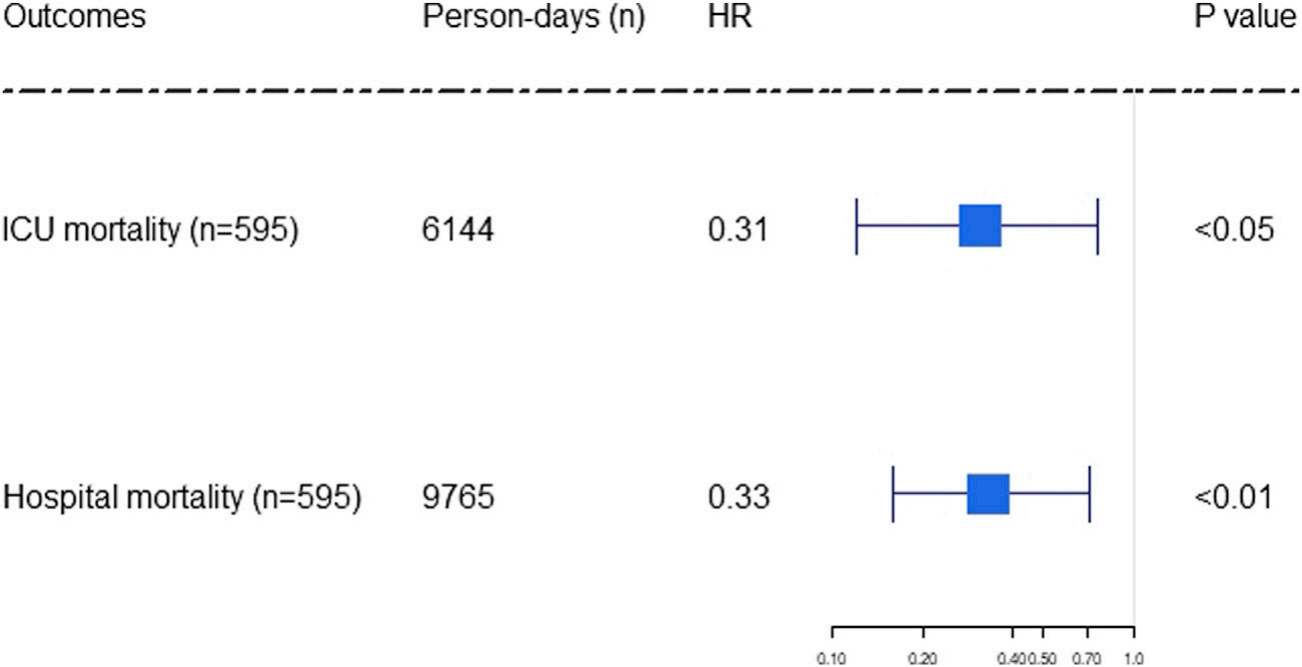
Forest plot showing the effect of sodium bicarbonate infusion on ICU and hospital mortality in elderly septic patients and acute moderate metabolic acidosis. The hazard ratios were estimated using the marginal structural Cox model. Person-days were the days of ICU and hospital length of stay. The *x*-axis tick marks follow a logarithmic scale. HR: hazard ratio.

Time-varying confounding factors and SB infusion were analyzed in the MSCM. The important variables of SB infusion for predicting hospital and ICU mortality were showed in Supplementary Tables S3, S4. MSCM results also revealed associations between SB infusion and reduced both ICU (HR 0.31, 95% CI 0.12–0.76; *p* < 0.05) and hospital (HR 0.33, 95% CI 0.16–0.71; *p* < 0.01) mortality in the subgroup with acute MMA (Figure 2, Supplementary Table S5).

## Discussion

The above findings in this study can be summarized as follows: (Vincent and Abraham, 2006): There was no association between SB infusion treatment and reduced ICU or hospital mortality overall in elderly septic patients with acute MA or in the subgroup of acute SMA; (Yahav et al., 2016); SB infusion was associated with reduced both ICU and hospital mortality for elderly septic patients in the subgroup of acute MMA. The current studies demonstrated that acute MA was frequently observed in the older critical patients with sepsis, and SB was commonly used in such patients but there was lack of evidence about its effect on mortality (Englert and Ross, 2015; Ganesh et al., 2016; Martin-Loeches et al., 2019; Fujii et al., 2021). Therefore, this is the first clinical investigation providing the evidence that SB infusion could improve both ICU and hospital survival for elderly septic patients with acute MMA.

SB has been widely administered in clinical practice treating acidosis in critically ill patients. However, there has not been well established evidence to support its effectiveness in septic patients with MA. A retrospective study suggested that septic patients with acidosis should be treated even more aggressively (Wernly et al., 2020). Another retrospective study revealed an association between SB and increased survival rates of sepsis in patients presented with AKI (stage 2 or 3) and severe acidosis (Zhang et al., 2018). The 2021 Surviving Sepsis Guideline suggested using SB for patients with septic shock, severe metabolic acidosis (pH ≤ 7.2) and AKI (stage 2 or 3 of AKIN score) (Evans et al., 2021), but there is insufficient evidence for elderly patients. The current study has shown that giving SB infusion is not potentially useful for elderly septic patients with acute SMA but is associated with improved outcomes for acute MMA. Despite that no relationship between SB infusion and improved mortality in the elderly septic patients with acute SMA was discovered in this study, SB infusion could still be administrated as a standard clinical practice.

As we anticipated, the most important predictors of SB infusion were PaCO2, pH and BC for elderly septic patients with acute MMA. There is also an association between the comorbidity of chronic kidney disease and SB treatment, because metabolic acidosis is a common condition among older adults who are diagnosed with chronic kidney disease. A clinical trial has revealed that oral SB is not beneficial to improve physical or renal function in older patients nor to reduce adverse events (BiCARB study group, 2020). Our study could provide supplemental evidence that SB infusion could be recommend for elderly septic patients with acute MMA, especially those with chronic kidney disease.

This study has some additional strengths. First, the MIMIC-IV database provides high-quality data record of a large cohort of elderly septic patients with acute MA to observe the potential linkage between SB infusion and mortality. Second, this investigation used PSA and MSCM to provide an explanation for baseline data and time-varying confounders. Third, this study further observed different subgroups and found meaningful evidence for acute MMA.

Some limitations of this study are also discussed. First, this selected cohort of patients were from a single institution while randomized controlled clinical trials might be more convincing. Second, some clinical information, such as fluid input and output were recorded incompletely due to unavailable data in the databases. Third, although PSA and MSCM were used balancing vital confounding variables, residual confounding could not be entirely prevented as the nature of the observational study design. Fourth, data in the MIMIC-IV were collected in a period of over 10 years during which clinical care had changed. Fifth, the missing data in the variables assessed in this study is a potential limitation. However, there was not any difference in baseline between the pooled imputed dataset and source dataset, and the analyses before (Supplementary Tables S6, S7) and after (Supplementary Tables S8, S9) multiple imputation for missing values yielded consistent results. Sixth, the SOFA score was not recorded and included in the variables assessed because of incorrect and missing data is another potential limitation. However, other correct and complete variables which were strongly associated with SOFA score assessment, such as comorbidities, the incidence of severe AKI and shock status and additional respiratory and hemodynamic support were all included into the analyses. Finally, the results yielded in this study also need further confirmation through rigorous clinical research such as randomized clinical trials with high-quality data.

## Conclusion

SB infusion treatment does not reduce ICU or hospital mortality of sepsis for elderly patients with acute MA overall or in the subgroup of acute SMA. However, it could improve both ICU and hospital survival in the subgroup of acute MMA. Further randomized controlled clinical trials are required to confirm our results.

## Data Availability

The original contributions presented in the study are included in the article/Supplementary Material, further inquiries can be directed to the corresponding authors.
