# Supplementary material for: Effectiveness of sodium bicarbonate infusion on mortality for elderly septic patients with acute metabolic acidosis
Source: Front Pharmacol. 2022 Sep 13;13:974271. doi: 10.3389/fphar.2022.974271 (PMC9513550; doi:10.3389/fphar.2022.974271)
Supplement: Supplementary file 1 [file DataSheet1.PDF]

Supplemental Tables of Contents

Supplemental Table 1: Information about data missingness.

Supplemental Table 2: Baseline differences between two groups after propensity score matching.

Supplemental Table 3: Predictors of the use of sodium bicarbonate infusion at each time point during ICU stay.

Supplemental Table 4: Predictors of the use of sodium bicarbonate infusion at each time point during hospital stay.

Supplemental Table 5: Results of marginal structural cox model for ICU and hospital mortality in older patients with sepsis and acute moderate metabolic acidosis.

Supplemental Table 6: Full multivariable model assessing impact of sodium bicarbonate infusion on ICU mortality in the overall and subgroups before multiple imputation.

Supplemental Table 7: Full multivariable model assessing impact of sodium bicarbonate infusion on hospital mortality in the overall and subgroups before multiple imputation.

Supplemental Table 8: Full multivariable model assessing impact of sodium bicarbonate infusion on ICU mortality in the overall and subgroups after multiple imputation.

Supplemental Table 9: Full multivariable model assessing impact of sodium bicarbonate infusion on hospital mortality in the overall and subgroups after multiple imputation.

Supplemental Table 1. Information about data missingness.

**Variables**	**Missing percentage, n(%)**
Cohort (n)	869
Age	0 (0)
Gender	0 (0)
BMI	26 (2.99)
Admission period	0 (0)
Sepsis, including pneumonia	0 (0)
Cardiovascular	0 (0)
Neurological	0 (0)
Other Respiratory	0 (0)
Other primary diseases	0 (0)
Hypertension	0 (0)
Diabetes	0 (0)
Congestive heart failure	0 (0)
Chronic pulmonary disease	0 (0)
Chronic kidney disease	0 (0)
Chronic liver disease	0 (0)
AKI	0 (0)
Renal replacement therapy	0 (0)
Shock	31 (3.57)
Mechanical ventilation	0 (0)
Vasopressors	0 (0)
Maximum PCO2	55 (6.33)
Minimum pH	17 (1.96)
Minimum bicarbonate concentration	0 (0)
Maximum lactate	39 (4.49)
Lactate solution	0 (0)
Use of sodium bicarbonate	0 (0)
Length of stay in ICU	0 (0)
Length of stay in hospital	0 (0)
ICU mortality	0 (0)
Hospital mortality	0 (0)

BMI: body mass index; AKI: acute kidney injury

Supplemental Table 2. Baseline differences between two groups after propensity score matching.

Key Characteristics	Non-SB group (n=)	SB group (n=)	P-value
**Demographic information:**			
Final cohort (n)	361	361	-
Age, years (median, (IQR))	75.00 (16.00)	73.00 (15.00)	0.06
Male gender (n(%))	180 (49.86)	185 (51.25)	0.77
BMI, (median, (IQR))	28.91 (9.15)	28.17 (8.61)	0.50
**Admission period, n(%)**			1.00
Before 2014	237 (65.65)	236 (65.37)	-
2014-2019	124 (34.35)	125 (34.63)	-
**Comorbidities (n(%)):**			
Hypertension	158 (43.77)	145 (40.17)	0.37
Diabetes	144 (39.89)	140 (38.78)	0.82
Congestive heart failure	139 (38.50)	128 (35.46)	0.44
Chronic pulmonary disease	61 (16.90)	50 (13.85)	0.30
Chronic kidney disease	126 (34.90)	129 (35.73)	0.88
Chronic liver disease	14 (3.88)	19 (5.26)	0.48
**The incidence of AKI stage 2 or 3 and shock status, (n(%)):**			
AKI-23	31 (8.59)	73(20.22)	<0.01
AKI-3	31 (8.59)	71 (19.67)	<0.01
AKI-2	0 (0)	2 (0.55)	0.48
Renal replacement therapy	34 (9.42)	87 (24.10)	<0.01
Shock	251 (69.53)	273 (75.62)	0.08
**Additional respiratory and hemodynamic support, (n(%)):**			
Mechanical ventilation	297 (82.27)	302 (83.66)	0.69
Vasopressors	300 (83.10)	315 (87.26)	0.14
**Laboratory values, (median, (IQR)):**			
Minimum PaO2, %	79.00 (29.00)	74.00 (30.00)	0.01
Maximum PaCO2, %	43.00 (10.00)	43.00 (14.00)	0.51
Minimum pH	7.21 (0.09)	7.16 (0.14)	<0.01
Minimum bicarbonate concentration, mmol/L	16.00 (4.00)	13.00 (5.00)	<0.01
Maximum lactate, mmol/L	3.60 (3.20)	5.80 (7.00)	<0.01
**Lactate solution, (n(%))**	154 (42.66)	142 (39.34)	0.41

AKI: acute kidney injury; AKI-2: AKI stage 2, AKI-3: AKI stage 3, AKI-23: AKI stage 2 or 3. AKI stages were based on serum creatinine criteria of KDIGO (Kidney Disease: Improving Global Outcomes).

Supplemental Table 3. Predictors of the use of sodium bicarbonate infusion at each time point during ICU stay

Key Characteristics	OR	Lower.95	Upper.95	P value
Demographic information:				
Age with 10 years increase	1.07	0.92	1.24	0.38
Gender (female as reference)	1.01	0.77	1.32	0.95
BMI with 5 increase	1.01	0.96	1.07	0.61
Admission period (before 2014 as reference)	0.54	0.38	0.76	<0.01
Comorbidities (n(%)):				
Hypertension	0.54	0.39	0.75	<0.01
Diabetes	1.13	0.86	1.49	0.38
Congestive heart failure	1.10	0.83	1.45	0.52
Chronic pulmonary disease	0.71	0.47	1.08	0.11
Chronic kidney disease	0.67	0.47	0.95	<0.05
Chronic liver disease	0.81	0.42	1.57	0.53
The incidence of severe AKI and shock status				
AKI-23	1.44	0.95	2.18	0.08
Renal replacement therapy	0.99	0.66	1.48	0.96
Shock	1.17	0.87	1.55	0.30
Additional respiratory and hemodynamic support, (n(%)):				
Mechanical ventilation	0.77	0.53	1.13	0.18
Vasopressors	1.21	0.84	1.74	0.31
Laboratory values, (median, (IQR)):				
PaCO2(mmHg) with 10 increase	0.68	0.57	0.82	<0.01
pH with 0.1 increase	0.42	0.33	0.53	<0.01
Bicarbonate (mmol/L) with 5 increase	0.33	0.25	0.43	<0.01
Lactate (mmol/L) with 0.1 increase	1.00	1.00	1.01	0.12
Lactate solution (L) with 1 increase	0.94	0.89	0.99	<0.05

BMI: body mass index; AKI: acute kidney injury; AKI-23: AKI stage 2 or 3. AKI stages were based on serum creatinine criteria of KDIGO (Kidney Disease: Improving Global Outcomes).

Supplemental Table 4. Predictors of the use of sodium bicarbonate infusion at each time point during hospital stay

Key Characteristics	OR	Lower.95	Upper.95	P value
Demographic information:				
Age with 10 years increase	1.02	0.89	1.17	0.75
Gender (female as reference)	1.08	0.84	1.38	0.54
BMI with 5 increase	1.01	0.96	1.07	0.73
Admission period (before 2014 as reference)	0.50	0.37	0.68	<0.01
Comorbidities (n(%)):				
Hypertension	0.61	0.45	0.82	<0.01
Diabetes	1.11	0.86	1.43	0.42
Congestive heart failure	1.32	1.03	1.70	<0.05
Chronic pulmonary disease	0.83	0.58	1.20	0.32
Chronic kidney disease	0.68	0.49	0.94	<0.05
Chronic liver disease	0.75	0.40	1.41	0.37
Primary diagnosis (n(%)):				
Sepsis, including pneumonia	0.75	0.52	1.10	0.14
Cardiovascular	0.91	0.63	1.31	0.62
Other Respiratory	0.77	0.55	1.07	0.12
Neurological	0.90	0.54	1.50	0.68
Others	0.92	0.60	1.41	0.70
The incidence of severe AKI and shock status				
AKI-23	1.40	1.97	2.03	0.07
RRT	1.11	0.78	1.58	0.58
Shock	1.12	0.87	1.46	0.38
Additional respiratory and hemodynamic support, (n(%)):				
Mechanical ventilation	0.80	0.57	1.13	0.20
Vasopressors	1.12	0.81	1.55	0.49
Laboratory values, (median, (IQR)):				
PCO2(mmHg) with 10 increase	0.74	0.64	0.85	<0.01
pH with 0.1 increase	0.49	0.41	0.59	<0.01
Bicarbonate (mmol/L) with 5 increase	0.32	0.26	0.40	<0.01
Lactate (mmol/L) with 0.1 increase	1.00	1.00	1.01	0.08
Lactate solution (L) with 1 increase	0.93	0.87	0.99	<0.05

BMI: body mass index; AKI: acute kidney injury; AKI-23: AKI stage 2 or 3. AKI stages were based on serum creatinine criteria of KDIGO (Kidney Disease: Improving Global Outcomes).

Supplemental Table 5. Results of marginal structural cox model for ICU and hospital mortality in older patients with sepsis and acute moderate metabolic acidosis.

Outcomes	Number of patients	Number of person-days	HR	Lower.95	Upper.95	P value
ICU mortality	595	6144	0.31	0.12	0.76	<0.05
Hospital mortality	595	9765	0.33	0.16	0.71	<0.01

AKI: acute kidney injury; AKI-23: AKI stage 2 or 3. AKI stages were based on serum creatinine criteria of KDIGO (Kidney Disease: Improving Global Outcomes).

Supplemental Table 6. Full multivariable model assessing impact of sodium bicarbonate infusion on ICU mortality in the overall and subgroups before multiple imputation.

**Overall and subgroups**	Hazard ratio	Lower.95	Upper.95	P value
Overall population (n=869)	0.70	0.49	1.00	0.05
Severe metabolic acidosis (n=274)	1.05	0.54	2.01	0.89
Moderate metabolic acidosis (n=595)	0.62	0.39	0.99	<0.05

Supplemental Table 7. Full multivariable model assessing impact of sodium bicarbonate infusion on hospital mortality in the overall and subgroups before multiple imputation.

**Overall and subgroups**	Hazard ratio	Lower.95	Upper.95	P value
Overall population (n=869)	0.82	0.61	1.09	0.17
Severe metabolic acidosis (n=274)	1.16	0.66	2.03	0.60
Moderate metabolic acidosis (n=595)	0.69	0.48	1.01	0.05

Supplemental Table 8. Full multivariable model assessing impact of sodium bicarbonate infusion on ICU mortality in the overall and subgroups after multiple imputation.

**Overall and subgroups**	Hazard ratio	Lower.95	Upper.95	P value
Overall population (n=869)	0.82	0.62	1.09	0.18
Severe metabolic acidosis (n=274)	1.14	0.70	1.84	0.60
Moderate metabolic acidosis (n=595)	0.63	0.43	0.93	<0.05

Supplemental Table 9. Full multivariable model assessing impact of sodium bicarbonate infusion on hospital mortality in the overall and subgroups after multiple imputation.

**Overall and subgroups**	Hazard ratio	Lower.95	Upper.95	P value
Overall population (n=869)	0.95	0.75	1.20	0.66
Severe metabolic acidosis (n=274)	1.26	0.83	1.91	0.27
Moderate metabolic acidosis (n=595)	0.72	0.53	0.99	<0.05
